# Thiamine modulates metabolism of the phenylpropanoid pathway leading to enhanced resistance to *Plasmopara viticola* in grapevine

**DOI:** 10.1186/1471-2229-13-31

**Published:** 2013-02-26

**Authors:** Hatem Boubakri, Anne Poutaraud, Mohamed Ali Wahab, Celine Clayeux, Raymonde Baltenweck-Guyot, Damien Steyer, Christophe Marcic, Ahmed Mliki, Isabelle Soustre-Gacougnolle

**Affiliations:** 1 Laboratoire de Physiologie Moléculaire des Plantes, Centre de Biotechnologie de Borj-Cédria, 2050, Hammam Lif, Tunisie; 2 Unité Mixte de Recherche 1131, Université de Strasbourg /INRA-Colmar, 28 Rue de Herrlisheim, F68021, Colmar, France; 3 Technopole de Borj-Cédria-CWR, 2050, Hammam Lif, Tunisie; 4 Twistaroma, 28 Rue de Herrlisheim, 68021, Colmar, France; 5 Equipe de Chimie Analytique des Molécules BioActives, UMR 7178, IPHC, Faculté de Pharmacie, Université de Strasbourg, 74 route du Rhin, 67400, Illkirch, France; 6 Laboratoire Vigne, Biotechnologies et Environnement (LVBE, EA3991), Université de Haute Alsace, 33 rue de Herrlisheim, 68000, Colmar, France

**Keywords:** Thiamine, *Plasmopara viticola*, Stilbenes, Phenylpropanoid pathway genes, Lignin, Flavonoids, Grapevine, Induced resistance, Real-Time q-PCR, HPLC, UPLC-MS

## Abstract

**Background:**

Previously, we have reported the ability of thiamine (vitamin B1) to induce resistance against *Plasmopara viticola* in a susceptible grapevine cv. Chardonnay. However, mechanisms underlying vitamins, especially, thiamine-induced disease resistance in grapevine are still largely unknown. Here, we assessed whether thiamine could modulate phenylpropanoid pathway-derived phytoalexins in grapevine plants, as well as, the role of such secondary metabolites in thiamine-induced resistance process to *P*. *viticola*.

**Results:**

Our data show that thiamine treatment elicited the expression of phenylpropanoid pathway genes in grapevine plants. The expression of these genes correlated with an accumulation of stilbenes, phenolic compounds, flavonoids and lignin. Furthermore, the total anti-oxidant potential of thiamine-treaded plants was increased by 3.5-fold higher level as compared with untreated-control plants. Four phenolic compounds are responsible of 97% of the total anti-oxidant potential of thiamine-treated plants. Among these compounds, is the caftaric acid, belonging to the hydroxy-cinnamic acids family. This element contributed, by its own, by 20% of this total anti-oxidant potential. Epifluorescence microscopy analysis revealed a concomitant presence of unbranched-altered *P*. *viticola* mycelia and stilbenes production in the leaf mesophyll of thiamine-treated inoculated plants, suggesting that stilbenes are an important component of thiamine-induced resistance in grapevine.

**Conclusion:**

This work is the first to show the role of thiamine, as a vitamin, in the modulation of grapevine plant secondary metabolism contributing to an enhanced resistance to *P*. *viticola*, the most destructive fungal disease in vineyards.

## Background

Grapevine (*Vitis vinifera*) is one of the most important horticultural fruit crops cultivated in the world. Unfortunately, all cultivars are susceptible to several diseases; fungi and oomycetes are the major pathogens that compromise the cultivation and economic profit from this plant. Downy mildew caused by the obligate biotrophic oomycete *Plasmopara viticola*, is one of the most destructive grapevine diseases. This disease reduces fruit quality and yield, either by direct infection of berries or as a result of the reduction in photosynthesis and plant vigor caused by leaf infections [1]. The protection of grapevine cultivars against this pathogen requires multiple applications of chemicals, starting from bud burst until ripening. Nevertheless, the disease control by fungicides leads to the emergence of resistant strains of *P*. *viticola *[2], environmental pollution, and toxic residues on food. In the last decade, a new technology for disease control, which involves the induction of host-defense mechanisms, was developed as an alternative to chemical fungicides.

This activation of the plant’s own defense system, known as induced resistance (IR), could be achieved by the application of inducers that mimic pathogen invasion [3]. IR in grapevine against *P*. *viticola* is characterized by the generation of H_2_O_2 _[4], enhanced expression of pathogenesis-related (PR) proteins with antimicrobial activity, such as chitinases and glucanases [4,5], phytoalexin production [6,7], and callose deposition [4,5].

Recently, the involvement of the phenylpropanoid pathway in IR mechanisms in grapevine was shown using a pharmacological approach [5]. Phenylpropanoid pathway-derived defense responses such as the synthesis of flavonoids, lignin, stilbenes and phenols have been shown to be associated with β-aminobutyric acid (BABA)-IR to *P*. *viticola* in grapevine [5,6]. A marked expression of phenylpropanoid pathway-derived phytoalexins including different stilbenic forms has been also reported to be associated with IR to *P*. *viticola* in grapevine by using chitosan oligomers [7]. In addition, β-1,3-Glucan-IR to *P*. *viticola* in grapevine was accompanied by a substantial accumulation of phenolic compounds [4], which are secondary metabolites that encompass several structurally diverse classes of natural products biogenetically arising from the phenylpropanoid pathway [8].

Phenolics constitute the main class of natural antioxidants present in plants and may function as reducing agents, free-radical scavengers, singlet oxygen quenchers, and potential complexers of pro-oxidants [9]. Phenolics seem to inhibit disease development via different mechanisms involving the inhibition of extracellular fungal enzymes (cellulases, pectinases, laccase, xylanase, etc.), inhibition of fungal oxidative phosphorylation, nutrient deprivation (formation of metal complexes, protein insolubilization), and antioxidant activity in plant tissues [10,11].

Low-molecular-mass secondary metabolites with antimicrobial activity that are induced by stress are collectively named phytoalexins, and are an important part of the plant defense repertoire. Phytoalexins are a heterogeneous group of compounds [12] that show biological activity towards a variety of pathogens and are considered as molecular markers of disease resistance.

Phytoalexins from the Vitaceae family have been the subject of numerous studies during the past decade, because these compounds are thought to have implications in both phytopathology and human health [13]. Although most phytoalexins are less phytotoxic than synthetic fungicides, they can accumulate in large quantities within plant tissues, far exceeding the concentrations necessary to inhibit fungal growth [13].

**Figure 1:**
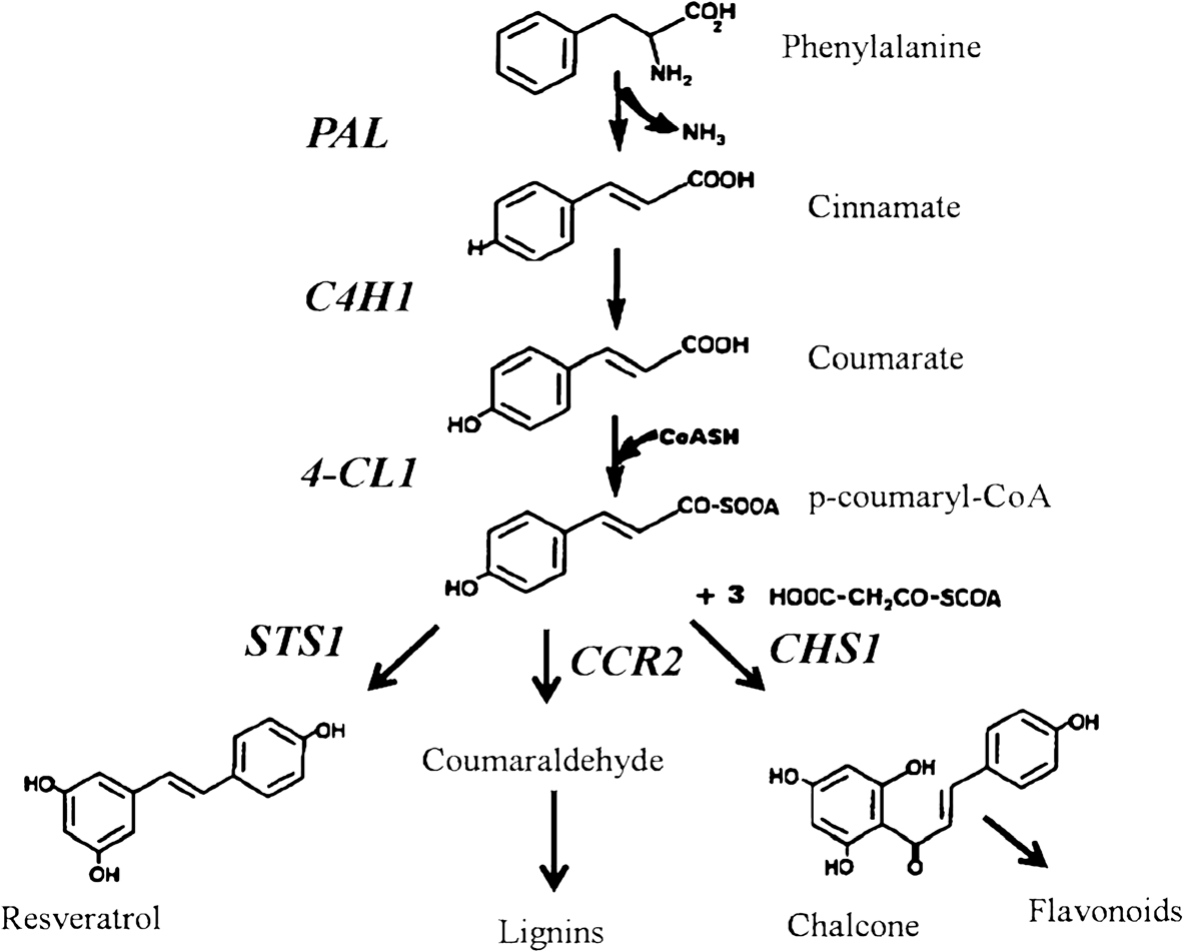
**Phenylpropanoid biosynthetic pathway modified according to Jeandet et al. **[13 ]**and Lijavetzky et al. **[14]**.** Genes encoding phenylpropanoid pathway enzymes are shaded in grey.

The general phenylpropanoid metabolism generates an array of secondary metabolites, which are based on the few intermediates of the shikimate pathway as the core unit [6]. The more relevant Vitaceae phytoalexins comprise a group of molecules belonging to the stilbene family [13]. Stilbenes are synthesized via the phenylpropanoid/malonate pathway from phenylalanine that, in turn, is converted into cinnamic acid by phenylalanine ammonialyase (*PAL*). The consecutive action of cinnamate 4-hydroxylase (*C4H*) and 4-coumarate CoA ligase (*4CL*) transform cinnamic acid into p-coumaryl-CoA. Compounds derived from this pathway, collectively referred to as polyphenols, are originated from this branching point through the action of the enzymes chalcone synthase (*CHS*), stilbene synthase (*STS*) and cinnamoyl-CoA reductase (*CCR*) for flavonoids, stilbenes, and lignin, respectively (Figure 1) [13,14].

Members of the stilbene family are derivatives of the *trans*-resveratrol structure (3,5,4^′^-trihydroxystilbene). Piceide (5,4^′^-dihydroxystilbene-3-O-β-glucopyranoside) is a glycosylated resveratrol derivative and pterostilbene (3,5-dimethoxy-4^′^-hydroxystilbene) is a dimethylated resveratrol derivative [13]. Recently, it was shown that an isomer of ε-viniferin, δ-viniferin, is one of the major stilbenes produced from resveratrol oxidation in grapevine leaves infected by *P*. *viticola *[15]. Pezet et al. [16] tested the toxicity of these stilbenes against zoospores of *P*. *viticola* (*in*-*vitro*) and found that δ-viniferin and pterostilbene were the most toxic stilbenes.

Currently, there is tremendous scientific and commercial interest in identifying chemicals whose exogenous application would activate plant defenses and afford protection from pathogen infection.

Elicitors induce production of phytoalexins by mimicking a pathogen attack or other stress [3], and can be substances of pathogenic origin (exogenous) or compounds released by the plants in response to the action of a pathogen (endogenous).

Inducers have potential uses in sustainable crop production. As critical components of many physiological processes, vitamins may influence the outcome of plant-pathogen interactions. In recent years, the importance of vitamins as nutrients and as control agents for different diseases has been demonstrated [17,18]. Ahn et al. [17] have reported that thiamine (vitamin B1) functions as an activator of plant disease resistance. Thiamine-treated rice, *Arabidopsis thaliana*, and vegetable crops showed resistance to fungal, bacterial, and viral infections. Thiamine-IR in rice and Arabidopsis plants was accompanied with a transient expression of *PR* genes through the salicylic acid- and Ca^2+^-related signaling pathways. In a previous study, we have reported the ability of thiamine to induce resistance against *P*. *viticola* in a susceptible grapevine cultivar “Chardonnay” by a dual mode of action involving direct antifungal activity and elicitation of host-defense responses including H_2_O_2_ generation, upregulation of *PR* genes, and hypersensitive cell death [19]. However, the mechanisms underlying vitamin-IR, and especially thiamine-IR, in grapevine are poorly known. In this study, we investigated the role of phenylpropanoid pathway metabolism in thiamine-IR to *P*. *viticola* in grapevine. Our experiments using real-time quantitative polymerase chain reaction (Real-Time q-PCR) demonstrated that phenylpropanoid pathway genes were upregulated by thiamine treatment. In addition, qualitative and quantitative analysis using high-performance liquid chromatography-diode array detection (HPLC-DAD), ultra-performance liquid chromatography coupled with mass spectrometry (UPLC-MS), chromatographic online antioxidant detection system (COADS), and histochemical analyses revealed that phenylpropanoid-derived phytoalexins such as flavonoids, phenols, lignin, and stilbenes were efficiently induced following treatment of grapevine plants with thiamine. Furthermore, epifluorescence microscopy observations suggested the possible involvement of stilbenes in the restriction of *P*. *viticola* mycelial growth in the leaf mesophyll of thiamine-treated grapevine plants.

## Results

### Effect of thiamine treatment on downy mildew incidence

**Figure 2:**
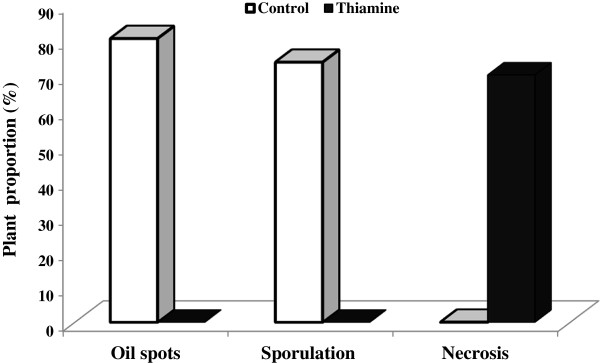
**Disease incidence (mean % of plants with visible symptoms) on grapevine cuttings treated with thiamine (solid bars) or water (white bars).** Grapevine plants grown under glasshouse, controlled conditions were treated with water (control) or 30 mM thiamine and the abaxial leaf surfaces were inoculated with a sporangial suspension at 5 × 10^4^ sporangia/ml. Disease incidence was assessed 7 dpi as proportion of plants showing necrosis, oil spots, and sporulation symptoms [20]. Six plants were used per treatment and the experiment was repeated 3 times. All figures in thiamine treatments are significantly different at 5% level from the controls.

Results shown in Figure 2 correspond to disease incidence on grapevine plants as mean % of plants with visible symptoms according to Unger et al. [20]. This data indicated that in control conditions, the average of plants showing pathogen sporulation was 63.4%, the average of plants showing pathogen oil spots was 70.3%, and the average of plants showing necrosis was 0%. In contrast, in the case of thiamine treatment, the average of plants showing pathogen sporulation symptoms was 0%, the average of plants showing pathogen oil spots was 0%, and the average of plants showing necrosis was 70%. Collectively, this data confirms the efficiency of thiamine in downy mildew control in grapevine plants.

### Thiamine upregulated phenylpropanoid pathway gene expression

**Figure 3:**
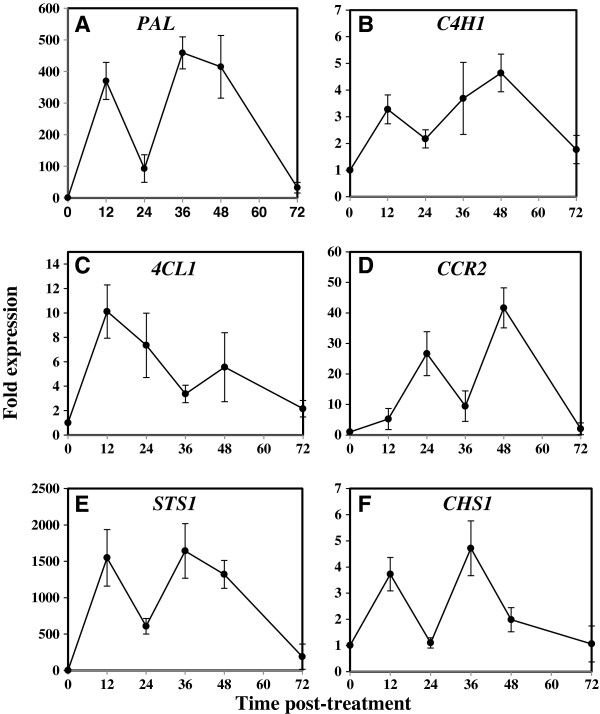
**Time course of transcript accumulation of genes of the phenylpropanoid pathway after thiamine treatment.** Grapevine plants (cv. Chardonnay) were treated with 30 mM thiamine (solid bars) or water (white bars) and leaves were collected at different time intervals post-treatment (12, 24, 36, 48, and 72 hpt). Gene expression was analyzed by real-time q-PCR. Genes of interest are: (**A**) *PAL* (phenylalanine ammonialyase), (**B**) *C4H1* (cinnamate 4-hydroxylase), (**C**) *4CL1* (4-coumarate:coenzyme A ligase), (**D**) *CCR2* (cinnamoyl:coenzyme A reductase), (**E**) *CHS1* (chalcone synthase) and (**F**) *STS1* (stilbene synthase1). Results are presented as normalized fold-expression of defense genes in thiamine-treated samples compared to expression in water-treated controls at the same time point, which is set to 1. Results are mean ± standard deviations of 3 independent experiments.

The data obtained (Figure 3A) showed that thiamine treatment induced a rapid and strong accumulation of *PAL* mRNA transcripts in grapevine plant leaves. The *PAL* induction by thiamine peaked at 2 time points. The first peak was detected within 12 hours post-treatment (hpt) with a 370-fold expression, whereas the second peak was observed at 36 hpt (459.11-fold expression) before decreasing progressively till 72 hpt (32.62-fold expression).

**Figure 4:**
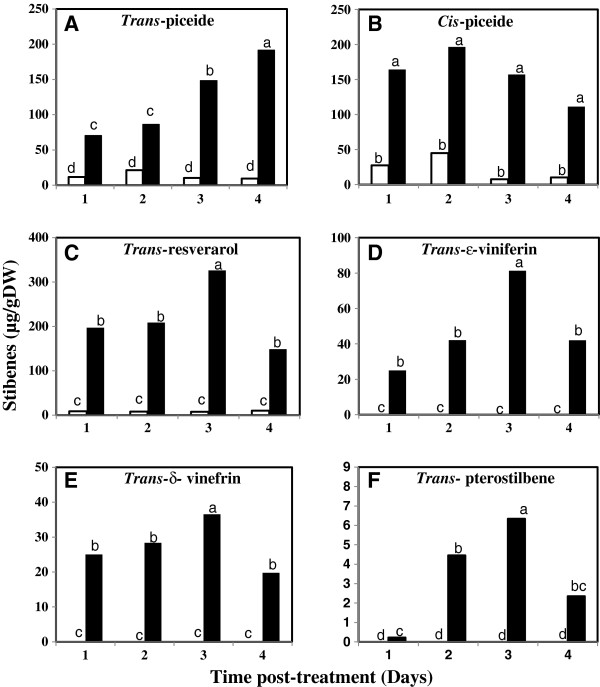
**Quantitative estimation of stilbenes in grapevine plants by HPLC-DAD.** Grapevine plants (cv. Chardonnay) were treated with 30 mM thiamine (solid bars) or water (white bars) and leaves were collected at different time intervals post-treatment (1, 2, 3, and 4 dpt) for methanolic extraction of stilbenes. Data are the mean of 3 independent experiments. (**A**) Trans-piceide, (**B**) Cis-piceide, (**C**) Trans-resveratrol, (**D**) Trans-ϵ-viniferin, (**E**)Trans-δ-viniferin and (**F**) Trans-pterostilbene.

*C4H1* (Figure 3B) and *4CL1* (Figure 3C) mRNA transcripts were also induced by thiamine treatment showing similar kinetics. In fact, both *C4H1* and *4CL1* inductions peaked first time within 12 hpt (3.27- and 10.11-fold expressions, respectively) and next time within 48 hpt (4.64 and 5.56-fold expression, respectively) before declining progressively till 72 hpt (1.77- and 2.15-fold expression).

Thiamine treatment also upregulated the expression of *CCR2* gene (Figure 3D) in grapevine plants. The CCR2 induction peaked the first time within 24 hpt (26.67-fold expression) and the second time within 48 hpt (41.65-fold expression) before being decreased at 72 hpt (2.01-fold expression).

Both *STS1* (Figure 3E) and *CHS1* (Figure 3F) genes were induced by thiamine treatment following a similar kinetic profile as that of the *PAL* gene. Indeed, *STS1* and *CHS1* mRNA transcripts peaked the first time at 12 hpt (1548 and 3.72-fold expression, respectively) and a second time within 36 hpt (1641.5- and 4.715-fold expressions, respectively). Subsequently, *STS1* and *CHS1* gene expressions decreased progressively until 72 hpt (188.14- and 1.06-fold expressions, respectively). Collectively, these results clearly demonstrate the ability of thiamine to elicit grapevine plants for an enhanced expression of phenylpropanoid pathway genes.

### Thiamine induced production of stilbenes

**Figure 5:**
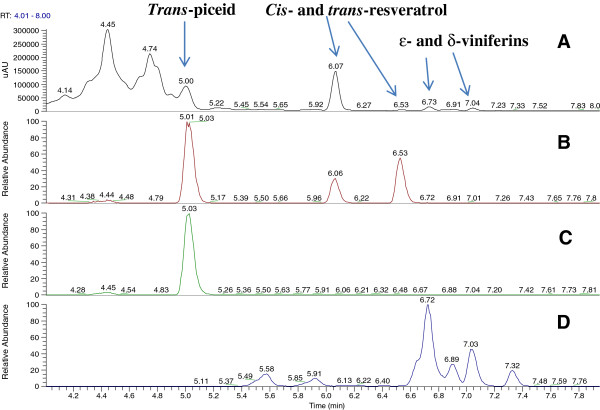
**Confirmation of the identity of stilbenoids by LC-UV-ESIMS(−) in methanolic extract of thiamine-treated plants (partial chromatogram).** (**A**) UV chromatogram at 307 nm, which permits the detection of stilbene derivatives specifically. (**B**) Extracted Ion Chromatogram of m/z 227.0703 corresponding to resveratrol derivatives (mass formula: C_14_H_11_O_3_). (**C**) Extracted Ion Chromatogram of m/z 389.1231 corresponding to piceide isomers (mass formula: C_20_H_21_O_8_). (**D**) Extracted Ion Chromatogram of m/z 453.1333 corresponding to viniferins (mass formula: C_28_H_21_O_6_).

To look for a correlation between *STS1* gene induction by thiamine treatment and stilbenes production, the amounts of different stilbenic compounds (stilbenoids) were determined using HPLC-DAD at different time intervals post-treatment (Figure 4). The identity of the different stilbenoids in samples used for HPLC-DAD analysis was also confirmed via ULPC-DAD-MS (Figure 5). Our results showed that the contents of both *trans*-piceide (Figure 4A) and *cis*-piceide (Figure 4B) were markedly elevated in thiamine-treated plants as compared to those of water-treated samples for the 4 investigated time points. The *trans*-piceide content increased with the increase in the time interval after thiamine application and reached its maximum level of 191.52 μg/g dry weight (DW) within 4 days post-treatment (dpt). However, the highest concentration of *cis*-piceide in thiamine-treated plants occurred at 2 dpt (196.52 μg/g DW). In contrast, lesser amounts of both *trans*- and *cis*-piceide were detected in water-treated samples at all the sampling times. *Trans*-resveratrol was quantitatively the most abundant stilbenoid produced after thiamine treatment (Figure 4C). *Trans*-resveratrol induction was detected starting from 1 dpt (197.6 μg/g DW), increased progressively with time and reached its maximum level at 3 dpt (326.23 μg/g DW) before decreasing at 4 dpt (148.76 μg/g DW). Only very meager amounts of *trans*-resveratrol were detected in water-treated plants at the different time points of sampling.

*Trans*-ε-viniferin (Figure 4D) and *trans*-δ-viniferin (Figure 4E) were detected only in thiamine-treated plants. Both *trans*-ε- and *trans*-δ-viniferins were produced starting from 1 dpt in thiamine-treated plants; thereafter, their content increased progressively reaching maximal levels within 3 dpt (81.34 and 36.56 μg/g DW, respectively) before decreasing at 4 dpt (42.03 and 19.78 μg/g DW, respectively). *Trans*-pterostilbene, like both *trans*-ε- and *trans*-δ-viniferins was not detected in water-treated plants at any of the time points of sampling. In contrast, it was significantly produced in thiamine-treated plants (Figure 4F) starting from 1 dpt. Then its content increased gradually with time, reaching maximum level at 3 dpt (6.34 μg/g DW) before decreasing within 4 dpt (2.35 μg/g DW).

### Thiamine treatment elicited the accumulation of phenols and lignin

The effect of thiamine on lignin and phenol synthesis was investigated using a histochemical approach. Grapevine plants were treated with water or thiamine and leaves were harvested at 1 dpt. For lignin detection, leaf discs were punched from water- and thiamine-treated plant leaves, stained with phloroglucinol, and examined under a light microscope. Red coloration of the leaf discs indicates the presence of lignin. A positive reaction, (red coloration) was observed in leaf discs of thiamine-treated plants (Figure 5A, C), indicating a marked induction of lignin synthesis in parenchymal tissues, whereas in the leaf discs of water-treated plants (Figure 5B), we did not observe any lignin accumulation.

For detection of phenols, leaf sections (10 μm in thickness) were obtained using a cryostat microtome and examined under an epifluorescence microscope. Production of phenols was observed as a blue coloration under UV-light. The leaf sections from thiamine-treated plants showed intense blue coloration, indicating accumulation of phenols (Figure 5D, F), unlike the leaf discs from water-treated plants (Figure 5E).

### Thiamine induced a prominent induction of flavonoids

**Figure 6:**
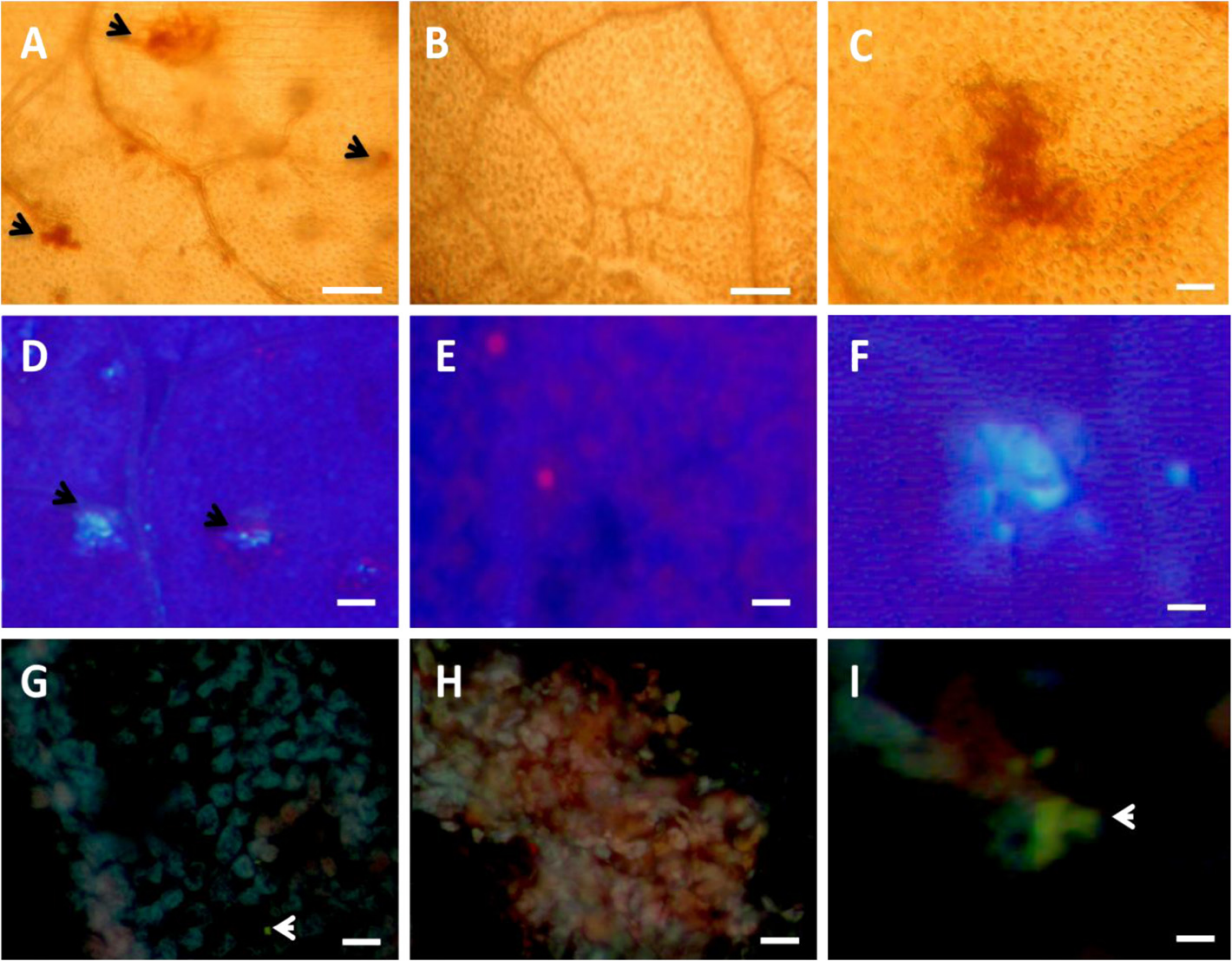
**Histochemical detection of lignin, phenolics, and flavonoids.** Leaves from plants treated with water (**B, E, G**) or 30 mM thiamine (**A, C, D, F, H, I**) were collected at 1 dpt, and used for microscopic studies. (**A, B, C**) Leaves from plants were used for subcellular detection of lignin accumulation as a red coloration under light microscope following phloroglucinol-HCl staining. (**D, E, F**) Leaf segments were examined under auto-fluorescence (UV light Filter); white–blue coloration indicates the presence of phenolics. (**G, H, I**) Leaf sections (10 μm of thickness) were stained with Wilson’s reagent and observed under UV light; yellow coloration indicates presence of flavonoids. Six leaves from different plants were assessed for each treatment and the whole experiment was repeated three times with similar results. Bars = 40 μM for (**A, B, D, E, G, H**) and 20 μm for (**C, F, I**).

Results of our molecular investigations have demonstrated that thiamine upregulated the expression of *CHS1* gene, which is responsible of flavonoid biosynthesis in grapevine. Therefore, we investigated whether the activation of this gene by thiamine correlated with an accumulation of flavonoids. The subcellular detection of flavonoids was achieved after staining with Wilson’s Reagent. In addition, we have assessed the amount of quercetin 3­*O*­glucoside (a flavonol) at different time points by HPLC-DAD. Our results (Figure 6H, I) showed that thiamine treatment induced a prominent accumulation of flavonoids evidenced by the presence of an intense yellow coloration after Wilson’s Reagent staining, whereas the intensity of this yellow coloration was markedly less evident in water-treated plants (Figure 6F).

**Figure 7:**
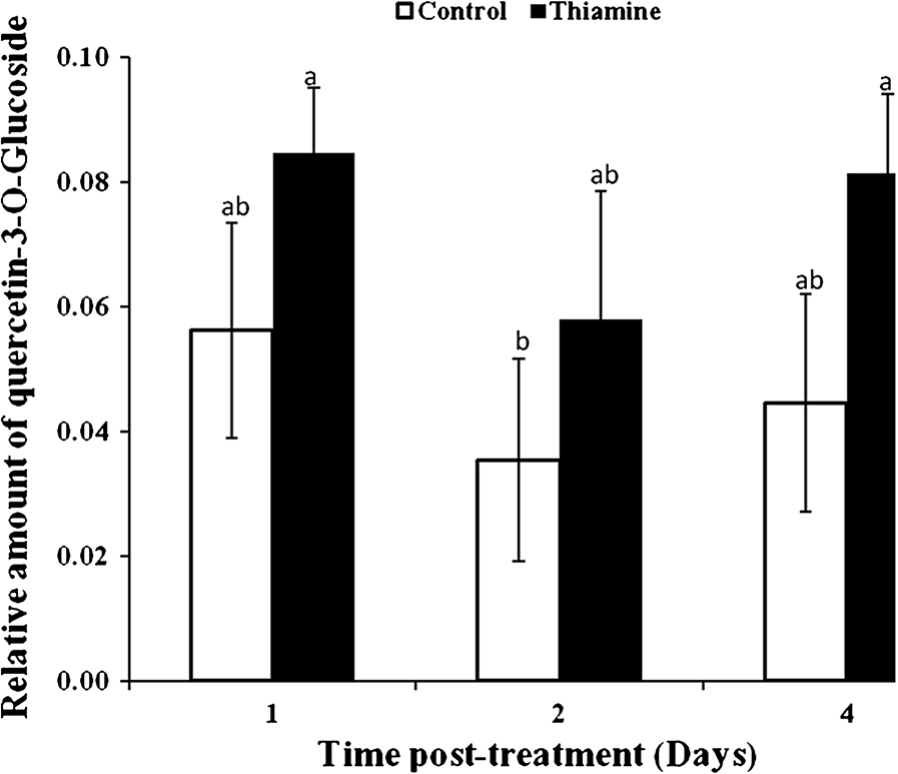
**Quantitative determination of quercetin 3-*O*-glucoside flavone in grapevine plants by HPLC-DAD.** Plants were treated with 30 mM thiamine (solid bars) or sterile distilled water (white bars) and leaves were harvested at different time intervals post-treatment (dpt). Data are the mean of 3 independents experiments.

The amount of quercetin 3-*O*-glucoside flavonol was not significantly affected by thiamine treatment of grapevine plants (Figure 7). Considering the large diversity of basal flavonoid structures in plants (flavones, dihydroflavonols, flavanones, isoflavonoids, isoflavans, and pterocarpans), other specific metabolites would be induced by thiamine.

### Thiamine enhanced the anti-oxidant potential of grapevine plants through induction of specific phenolic compounds, including a caftaric acid

**Figure 8:**
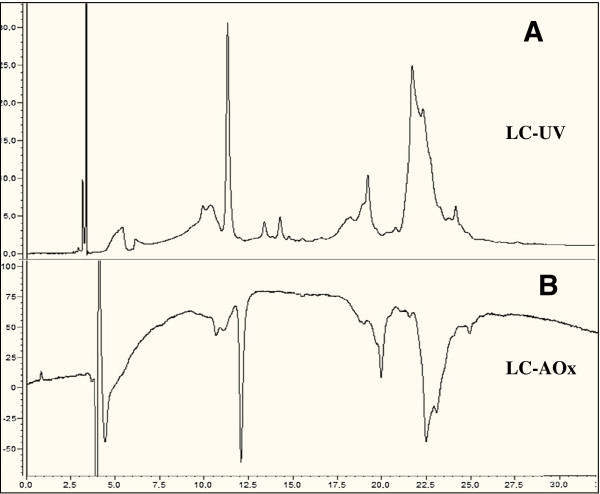
**Chromatographic determination of phenolic compounds (A) and their corresponding anti-oxidant activities (B) in extracts from thiamine-treated grapevine plants.** Detection was done at 280 nm for phenolic compounds (LC-UV) and at 412 nm for their respective antioxidant activities after post-column reaction with (ABTS[*][+]) (LC-AOx). The whole experiment was repeated 3 times with similar results.

**Figure 9:**
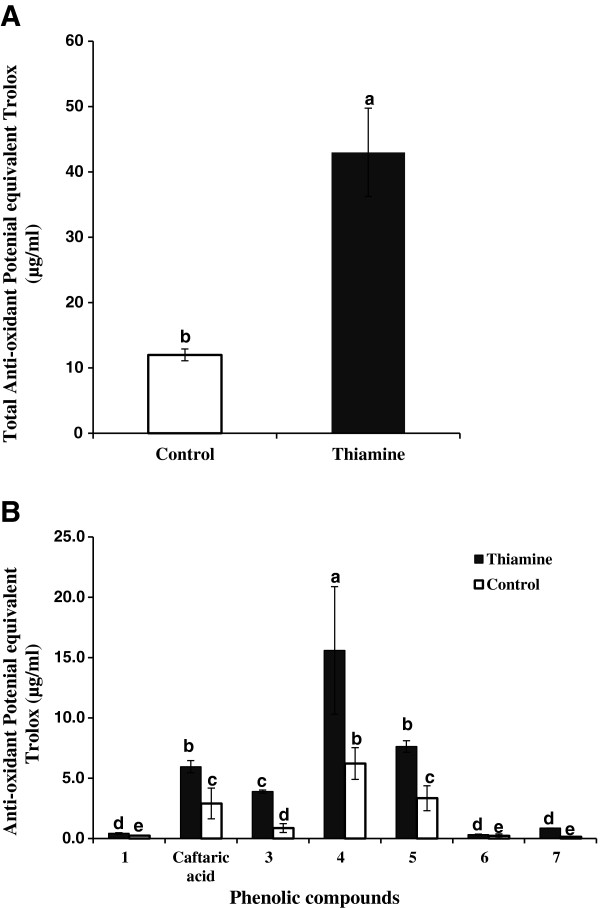
**Increase of the antioxidant potential of grapevine plants after thiamine treatment.** (**A**) COADS determination of the total anti-oxidant potential of leaf extracts from water (white bars) and thiamine-treated (solid bars) grapevine plants. (**B**) Individual contribution of phenolic compounds to the anti-oxidant potential of water (white bars) and thiamine-treated (solid bars) plants. Data are the mean of 3 independents experiments.

Figure 8 shows COADS chromatograms corresponding to the phenolic compounds separated in a methanolic extract of thiamine-treated grapevine plants and their corresponding antioxidant activities. The result in the upper part of the figure was obtained using direct UV detection at 254 nm, whereas that at the lower part was obtained using visible detection at 412 nm after post-column reaction. For each treatment, COADS chromatograms were determined and peak areas were summed and used to represent the total antioxidant activity, which were expressed as average ± SD from triplicates of 3 determinations (Figure 8A). The total antioxidant activity results were expressed as Trolox equivalent (μg/ml). The total antioxidant potential in thiamine-treated plants was 43 ± 6.8 μg/ml Trolox equivalent, whereas in water treated-plants, it was 12 ± 0.9 μg/ml, indicating that the total antioxidant potential of thiamine-treated plants was 3.58-fold that of control plants (Figure 9A). The largest antioxidant contribution came from only 4 compounds, which were responsible for 97% of the total antioxidant potential of thiamine-treated plants. Among them, we identified a caftaric acid belonging to the family of hydroxyl-cinnamic acids, which by itself contributes about 20% in the total antioxidant potential (Figure 9B). Work is under way to identify the other unknown phenolic compounds.

### A possible role for stilbenes in *P*. *viticola* restriction by thiamine

**Figure 10:**
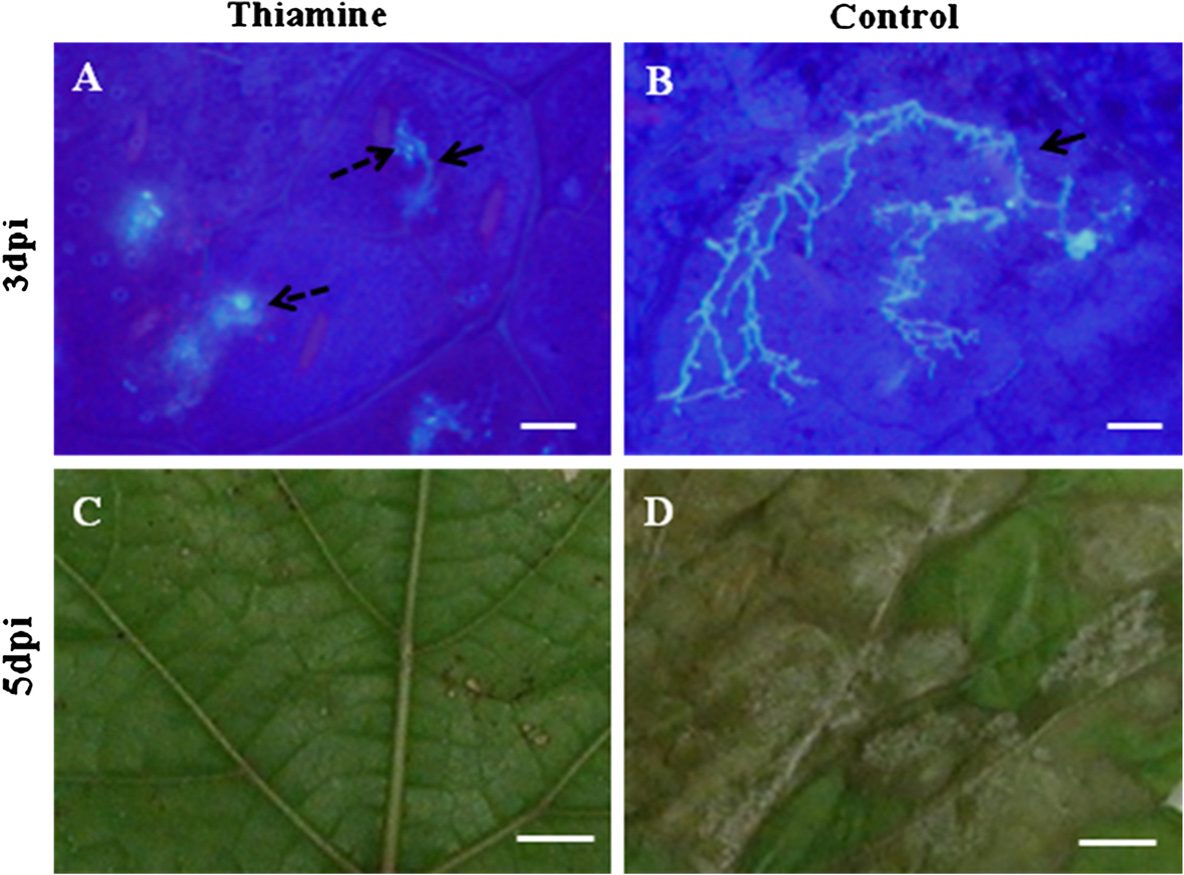
**A possible role for stilbenes in the restriction of *P*. *viticola *development by thiamine treatment.***Vitis vinifera* cv. Chardonnay plants were treated with water or thiamine for 24 h and inoculated by spraying *P*. *viticola* sporangia suspension (10^4^ sporangia/ml). (**A**) Presence of fluorescent material (discontinued arrows) under autofluorescence corresponding to stilbenes [6], surrounding unbranched and degenerated hyphae (discontinued arrows) of *P*. *viticola* in the mesophyll of thiamine-treated plant leaves. (**B**) Absence of stilbenes accumulation and extensive branching of *P*. *viticola* hyphae in the intercellular space of the mesophyll of water-treated plant leaves. (**C**) Development of necrotic zones (HR) and suppression of *P*. *viticola* sporulation in thiamine-treated plants. (**D**) Development of *P*. *viticola* sporulation symptoms in water-treated plants. Bars = 40 μM for (**A, B**) and 1 cm for (**C, D**).

Stilbenes have been reported to possess a biocidal effect against *P*. *viticola* zoospores *in vitro*[16]. Here, we assessed their effect on the mycelia growth of the pathogen in grapevine plants, elicited by thiamine (*in vivo*). Accumulation of both stilbenes and pathogen mycelia was detected in foliar tissues by epifluorescence microscopy (under UV light). The results (Figure 10), clearly showed the presence of altered (Figure 10A), unbranched pathogen mycelia inside foliar zones that showed intense blue colorations corresponding to the accumulation of stilbenes in thiamine-treated plant leaves. In contrast, in water-treated plants, we observed normal and extensive hyphae development in the leaf mesophyll, and we did not observe any stilbene accumulation (Figure 10B). Thus, the concomitant presence of altered, unbranched mycelia and stilbene production in thiamine-treated plant leaves suggests a possible contribution of this phytoalexin family to *P*. *viticola* restriction by inhibition of mycelia spreading in the leaf mesophyll. In addition, direct effects of thiamine might also play a role in the alteration of pathogen mycelia. At 5 days post-inoculation (dpi), we observed a heavy infection of *P*. *viticola* in the leaves of water-treated plants, evidenced by the development of white sporulation symptoms (Figure 10D), whereas in thiamine-treated samples, we observed only a development of necrotic zones corresponding to hypersensitive reaction related-cell death (Figure 10C).

## Discussion

The major phytoalexins produced by *V*. *vinifera* cultivars during pathogen infections are resveratrol and its derivatives, piceide (the glycosylated form), δ-viniferin and ε-viniferin (oxidised dimers), and pterostilbene (the methylated derivative) [21]. In our experiment, thiamine treatment led to an induction of *trans*- and *cis*-piceide forms with maximal levels obtained within 4 and 2 dpt, respectively for each compound. Thiamine treatment also induced the accumulation of *trans*-resveratrol with a maximal level observed within 3 dpt. In susceptible grapevine cultivars, resveratrol is synthesized in large amounts, but it is rapidly glycosylated into the non-toxic compound piceide. This could explain the high concentrations of both *cis*- and *trans*-piceide in thiamine-treated plants, which is due to the high concentration of *trans*-resveratrol. Further, thiamine elicited the accumulation of important stilbenic forms, the viniferins, which are active against *P*. *viticola*; δ-viniferin is 5 times as toxic as ε-viniferin [16]. The maximal levels of both ε- and δ-viniferin were observed at 3 dpt. Both ε- and δ-viniferin were not present in water-treated plants. The increase in resveratrol synthesis at 3 dpt for thiamine-treated plants provided an important pool for the synthesis of viniferins that also occurred at 3 dpt. In addition, thiamine treatment induced pterostilbene synthesis with a maximal induction level observed also at 3 dpt.

Pterostilbene was found to be very effective as a lipid/lipoprotein-lowering agent in hypercholesterolemic hamsters, probably because, like fibrate drugs, pterostilbene was shown to act as a peroxisome proliferator-activated receptor agonist [22]. Moreover, pterostilbene exhibits antioxidant and anticancer properties similar to those of resveratrol [23]. Pterostilbene was found to be very effective in preventing carcinogen-induced preneoplastic lesions in a mouse mammary organ culture model [24], and it showed preventive activity against colon carcinogenesis in rats [25].

Flavonols are phenylpropanoid metabolites, most of which are synthesized from p-coumaroyl-CoA and malonyl-CoA and share their precursors with the biosynthetic pathway for lignin biosynthesis [26]. However, some rare flavonoids are synthesized from CoA esters of substrates such as cinnamic acid or dihydrocoumaric acid. Flavonoids also contribute to resistance against pathogens. In our experiment, thiamine induced the accumulation of flavonoids. In contrast, such metabolic response is absent in all susceptible grapevine cultivars after pathogen inoculation [27]. Many specific flavonoids belonging to flavone and flavanone classes have been shown to be active against fungal pathogens commonly found during the storage of fruits and vegetables, i.e. *Aspergillus* sp., *Botrytis cinerea* and *Fusarium oxysporum *[8]. Flavonoids have also been reported to be involved in the restriction of *P*. *viticola* growth in intermediate resistant species *Vitis rotundifolia* and *Vitis rupestris*[28].

A part of phytoalexins functions as antioxidant and thereby contribute to plant resistance against pathogens [29]. In our study, thiamine application induced a prominent accumulation of specific phenolic compounds with antioxidant properties, which led to an increase in the total antioxidant potential of grapevine plants as demonstrated by COADS analysis. Previous studies have demonstrated that, in resistant plants, cells usually respond by increasing the level of pre-existing antifungal phenols at the infection site, after an elicited increased activity of the key enzymes (PAL and CHS) of the phenylpropanoid pathway [30]. Phenolic esterification in cell wall materials is usually considered as an increase in resistance to fungal hydrolytic enzymes as well as a physical barrier against fungal penetration [11]. The first demonstrated example of phenolics providing disease resistance was the case of onion scales accumulating sufficient quantities of catechol (I) and protocatechuic acid (II) to prevent onion smudge disease, *Colletotrichum circinans*. The colored outer onion scales of resistant onion varieties contain enough of these 2 phenols to reduce spore germination of *C*. *circinans* to below 2%, whereas in susceptible varieties, which lack these compounds, the germination rate is over 90% [30]. Adequate levels of chlorogenic acid (III) account for the resistance of potato tubers against *Phytophthora infestans *[30]. Phenolics have also been reported to be involved in the restriction of *P*. *viticola* growth in intermediate resistant species *V*. *rotundifolia* and *V*. *rupestris*[28]. In contrast, such metabolic response is absent in susceptible grapevine cultivars after pathogen inoculation [27]. In addition, accumulation of phenolics has been reported to be associated with IR mechanisms in grapevine, such as β-1,3-glucan- and chitosan-IR to *P*. *viticola*[4,7].

On the other hand, the increased level of phenolics may provide an adequate substrate for oxidative reactions catalyzed by polyphenol oxidase (PPO) and/or peroxidase (POD), which, by consuming oxygen and producing fungitoxic quinones, make the medium unfavorable to the further development of pathogens [8]. Vanci et al. [31] have reported that peroxidase activity using specific phenolic compounds as substrate lead to lignin formation.

Our study revealed enhanced accumulation of phenolics and lignin in thiamine-treated grapevine plants. These compounds have been reported to play a key role in limiting fungal extension [32]. In addition, Cohen et al. [33] assumed that lignin encasing cells of muskmelon invaded with haustoria of *Pseudoperenospora cubensis* prevent fungal growth and induced host cell death by interrupting the nutrient flow into and out of the cells. Taken together, these findings suggest that both phenolics and lignin might play a possible role in thiamine-IR to *P*. *viticola* in grapevine.

Previously [19], we have reported that a time interval of 3 days between thiamine treatment and pathogen inoculation is the optimal time for a maximal resistance to *P*. *viticola*. In the present work, we found that the maximal induction of specific stilbenes (both ε- and δ-viniferin and pterostilbene) by thiamine treatment also occurred at 3 dpt. Thus, these results might indicate a close correlation between stilbene accumulation and IR to *P*. *viticola* by thiamine treatment. Furthermore, our microscopic observations clearly demonstrated a concomitant presence of altered, unbranched *P*. *viticola* mycelia and stilbene accumulation in thiamine-treated plants. Collectively, these results indicate a possible contribution of stilbenes in thiamine-IR to *P*. *viticola* in grapevine.

## Conclusion

As a conclusion, the findings denoted in this paper emphasized the role of phenylpropanoid pathway metabolism in thiamine-IR to *P*. *viticola* in grapevine. In fact, thiamine application upregulated phenylpropanoid pathway gene expression and elicited the plants to accumulate specific stilbenes like both ε- and δ-viniferins and pterostilbene, that are otherwise not present in the plants. Moreover, thiamine induced the accumulation of phenolics, lignin, and flavonoids, which are markers of resistance to *P*. *viticola* in intermediate resistant grapevine species. As a result of these molecular and cellular changes, the antioxidant potential of grapevine plants was increased, which could provide additional mechanisms contributing in downy mildew restriction by thiamine. Furthermore, we found a concomitant presence of phytoalexins-derived stilbenes and degenerated pathogen mycelial structures in the leaf mesophyll of thiamine-treated plants, suggesting a possible involvement of stilbenes in thiamine-IR to *P*. *viticola* in grapevine. Therefore, we could suggest that the induction of phenylpropanoid pathway-derived phytoalexins by thiamine might be valuable in developing alternative strategies to chemical fungicides for controlling downy mildew in vineyards. Moreover, this finding would open the door to a large community of *vitro* culturists, who extensively studied the possible use of plant cultures for the production of secondary compounds of industrial interest (mainly pharmaceutics and dyes).

## Methods

### Plant material and growth conditions

*Vitis vinifera* “Chardonnay cv” plants were obtained from herbaceous cuttings. They were cultivated in pots containing a mixture of peat and perlite (4:1, v/v). Plants were grown under controlled glasshouse conditions (24°C, 16 h light and 8 h dark photoperiod and 70% RH) until they developed 11 leaves. Plants were watered every 2 days with a nutritive solution (1 g/l) (Plant Prod 15-10-30, France).

### Pathogen

*Plasmopara viticola* sporangia [34], were propagated on *V*. *vinifera* cv. Chardonnay detached leaves maintained in sealed Petri dishes on humid Whatman 3MM paper. Abaxial leaf surfaces were sprayed with freshly collected sporangia re-suspended in water at 5 × 10^4^ sporangia/ml. Inoculated leaves were placed in a growth chamber at 20°C and 100% RH for 24 h in the dark, then under a 16 h light and 8 h dark photoperiod and 70% RH for 6 days. Inoculum of *P*. *viticola* sporangia was prepared by washing the lower side of grapevine leaves that carried freshly sporulating lesions with distilled water. The sporangia suspension was then adjusted to a concentration of 10^4^ sporangia/ml after counting with a haemocytometer under a light microscope.

### Treatments

Grapevine plants were treated with 30 mM thiamine or water (control) on both upper and lower leaf surfaces until the point of run-off using a compressed air hand-sprayer device. Treated plants were kept in a growth chamber at 25°C, a 16 h light and 8 h dark photoperiod, and 70% RH.

### Pathogen inoculation procedure and determination of disease incidence

Grapevine plants grown under glasshouse, controlled conditions were treated with water (control) or 30 mM thiamine and the abaxial leaf surfaces were inoculated with a sporangial suspension at 5 × 10^4^ sporangia/ml using a compressed-air hand sprayer device. Inoculated plants were incubated overnight in dark at 80% RH and 20°C and then kept in a growth chamber under controlled conditions at 24°C, 16 h light and 8 h dark photoperiod, and 70% RH. At 5 dpi, plants were incubated overnight in darkness at 80% RH and 20°C to allow downy mildew sporulation and disease incidence was assessed as proportion of plants showing necrosis, oil spots, and sporulation symptoms [20]. Six plants were used per treatment and the experiment was repeated 3 times.

### RNA extraction and reverse transcription (RT)

Total RNA was isolated from grapevine plant leaves by using the RNeasy plant mini kit (Qiagen, Germany) according to the manufacturer’s instructions and quantified using a Nanodrop ND-1000 spectrophotometer (Thermo Scientific, Waltham, MA). Residual genomic DNA was removed by performing on-column DNase I digestion by using the RNase-Free DNase set (Qiagen). Total RNA (500 ng) was used as the template for RT by using the SuperScript II Reverse Transcriptase (Invitrogen, Carlsbad, CA) and oligodT-18 as recommended by the supplier.

### Real-Time Quantitative Polymerase Chain Reaction (RT q-PCR)

The expression pattern of phenylpropanoid pathway genes (Table 1) was determined in grapevine cultivar Chardonnay plants after thiamine treatment. RT q-PCR was performed using the absolute qPCR SYBR Green Mix (Eurogentec, Belgium) in 96-well plates in a volume of 25 μl in a buffer containing 1× SYBR Green Mix (including *Taq* polymerase, dNTP, and SYBR Green dye), 100 nM primers and 5-fold diluted reverse-transcribed RNA. PCR conditions were 5 min at 95°C followed by 40 cycles, each consisting of a step of denaturation (20 s at 95°C), a step of hybridization (30 s at 55°C), and a step of annealing (1 min at 60°C). To check the specificity of the PCR product, melting curves were analyzed for each data point. The absence of primer dimer formation was checked in controls without template. The kinetic of transcript levels were determined with ubiquitin gene (Ubq) as internal control. Sequences of the primers for Ubq (accession No.TC32075) are 5^′^­GTGGTATTATTGAGCCATCCTT­3^′^ for forward reaction and 5^′^­AACCTCCAATCCAGTCATCTAC­3^′^ for reverse. Each time point was determined as an average of 3 independent experiments. Relative gene expression was determined using the formula, fold induction = 2^–ΔΔCt^, where ΔΔC_T_ = (C_T_ GI [unknown sample] – C_T_ GI [reference sample]) – (C_T_ Ubq [unknown sample] – C_T_ Ubq [reference sample]) as previously reported by Trouvelot et al. [4]. GI is the gene of interest. The reference sample is the water-treated samples chosen to represent 1× expression of the gene of interest.

**Table 1:** **Primers used for the expression analysis of phenylpropanoid pathway genes by using RT q-PCR **[[14]]

**Gene abbreviation**	**Gene definition**	**Gen Bank accession**	**Unigene ID**	**Primer pair**
				**5**^**′**^**-Forward-3**^**′**^**/5**^**′**^**-Reverse-3**^**′**^
*STS1*	Stilbene synthase 1	DO366301	Vvi.8	CGAAGCAACTAGGCATGTGT/
				CTCCCCAATCCAATCCTTCA
*PAL1*	Phenylalanine ammonialyase	EC987386	Vvi.1950	CCGAACCGAATCAAGGACTG/
				GTTCCAGCCACTGAGACAAT
*C4H1*	Cinnamate-4-hydroxylase	EC995763	Vvi.6228	AAAGGGTGGGCAGTTCAGTT/
				GGGGGGTGAAAGGAAGATAT
*4CL1*	4-coumarate-CoA ligase	EC947790	Vvi.1251	CTGATGCCGCTGTTGTTTCG/
				GCAGGATTTTACCCGATGGA
*CHS1*	Chalcone synthase1	EC996578	Vvi.117	GTCCCAGGGTTGATTTCCAA/
				TCTCTTCCTTCAGACCCAGTT
*CCR2*	Cinnamoyl-CoA reductase	CF517687	Vvi.15864	ACAGCATGACGACTCTCTTCG/
				AGTGACAAGGGGTGGATTGA

### Chemical solvents used for HPLC-DAD and UPLC-MS analysis and their references

HPLC-MS grade acetonitrile and formic acid were supplied by Thermo Fisher Scientific; water was provided by a Millipore water purification system (Millipore, Bedford, MA). Reference compounds including *trans*-resveratrol, *trans*-piceide, *trans*-pterostilbene, and ε-viniferins were purchased or kindly provided by R. Pezet, Changins, Switzerland. The peak identity related to *trans*-resveratrol, *trans*-piceide, and *trans*-pterostilbene was verified by comparing the retention time, the UV, and the exact mass spectra of authentic standard.

### HPLC–DAD quantification of stilbenes

Grapevine cuttings grown under glasshouse conditions were treated with water (control) or thiamine (30 mM) and leaves on the fourth and fifth positions from the apex were harvested at various time intervals post-treatment, weighed, and used for methanolic extraction. Stilbene extractions and HPLC-DAD analysis were performed according to Poutaraud et al. [35]. Briefly, 800 μl of methanol was added to 160 mg (FW) of leaf tissues followed by incubation at 60°C for 45 min using a heating block, then the mixture was centrifuged twice at 12000 rpm for 10 min. Twenty microliter of the recuperated supernatant were injected in the HPLC-DAD system. The HPLC system consisted of a 1100 quaternary pump (Hewlett-Packard, Agilent Technologies, Massy, France) equipped with a 1100 photodiode array multiwavelength detector (Hewlett-Packard), a 1100 vacuum degasser (Hewlett-Packard), and a 234 automatic injection module (Gilson, Villiers-le-Bel, France). The analyses were carried out at 20°C on a Lichrospher end-capped RP-18 column (5 μm, 250 mm × 4.6 mm, Merck, Lyon, France). The absorption was measured between 200 and 400 nm, and chromatograms were recorded at 307 nm. Stilbenes were identified by comparing their retention times and their UV absorption spectra with those of the reference standards.

### UHPLC-MS conditions

The identity of each peak of stilbenes on leaf methanolic extracts analyzed by HPLC-DAD was confirmed using an Ultra High Performance Liquid Chromatography system (UHPLC, Ultimate 3000, Dionex, Thermofisher Scientific, San Jose, CA) equipped with a binary pump, an online degasser, a thermostated autosampler, a thermostatically controlled column compartment, and a diode array detector. The chromatographic separation was performed on an Agilent RRHD C18 SB column (150 × 2.1 mm, 2.7 μm particle size) maintained at 20°C. The mobile phase was composed of 0.1% formic acid in acetonitrile (solvent A) and 0.1% formic acid in water (solvent B) at a flow rate of 0.25 ml/min. The gradient elution program was: 0–1 min, 80% B; 1–11 min, 80–0% B; and 11–12 min, 0% B. The sample volume injected for each extract was 1 μl. The absorption was measured between 190 and 400 nm, and chromatograms were recorded at 280 and 307 nm. The liquid chromatography system was coupled to an Exactive Orbitrap mass spectrometer (Thermo Fischer Scientific).

The mass spectrometer consisted of an electrospray ionization (ESI) source operating in negative mode. Parameters were set at 300°C for ion-transfer capillary temperature and −2500 V needle voltage. Nebulization with nitrogen sheath gas and auxiliary gas were maintained at 40 and 6 arbitrary units, respectively. The instrument was operated at 50 000 resolution, and the spectra were acquiring within the m/z mass range of 100–1000 a.m.u. The instrument was operated using the program ExactiveTune, and data were processed using the XcaliburQual software.

The system was calibrated externally using the calibration mixture of Thermo Fischer Scientific in the scan range of m/z 100–2000 a.m.u. The calibration was performed every 7 days with MSCAL6-1EA. Such a calibration gave at least 2 ppm accuracy. The identification of stilbenes was confirmed by comparing their retention times and their mass spectra with those of the reference standards and by obtaining their pseudo molecular ion peak (M–H), which permitted the establishment of their precise molecular formula.

### Determination of the total antioxidant potential and phenolic compounds by using COADS

The grapevine plant extracts were screened for radical scavenging capacity of individual phenolic compounds, using an on-line HPLC post-column reaction assay [36]. The assay is based on the ability of radical scavenging compounds to reduce the green–blue-colored 2,2^′^-azinobis-3-ethylbenzothiazoline-6-sulfonic acid (ABTS[*][+]) radical cation into non-colored form. This results in a decrease of the absorbance at 412 nm. COADS chromatograms were determined for each treatment and peak areas were summed and used to represent the total antioxidant activity. The HPLC system (Ultimate 3000, Dionex, ThermoScientific, Gometz le Châtel, France) consists of a DGP 3600 Pump, a WPS-3000T Autosampler, a 3000 Diode Array Detector, and a 3100 Variable Wavelength Detector. A 3-m long and 0.25-m i.d. PEEK reaction coil was used. Data were acquired and processed using Dionex Chromeleon 6.8 data system.

Separation was carried out at room temperature on a Hypersil BDS C18 HPLC column (5 μm, 250 × 4.6 mm i.d., ThermoScientific, Gometz-le-Châtel, France). The mobile phase, delivered at 1 ml/min, consisted of a gradient mixture of water containing 0.1% formic acid (eluent A) and acetonitrile (eluent B). The following gradient was used: 0–25 min, 3–25% B; 25–29 min, 25% B; 29–37 min, 25–30% B; 37–47 min, 30% B; 47–57 min, 30–65% B; 57–67 min, 65% B. The (ABTS[*][+]) solution was delivered at 0.5 ml/min. Detection was carried out at 280 nm for phenolic compounds and at 412 nm for their respective antioxidant activities after post-column reaction with (ABTS[*][+]). The results are expressed in μg/ml Trolox equivalent, a water-soluble synthetic vitamin E derivative 6-hydroxy-2,5,7,8-tetramethylchroman-2-carboxylic acid, which was used as standard anti-oxidant.

### Histochemical detection of flavonoids, phenols, and lignin

Leaves from plants treated with water (control) or thiamine (30 mM) were used for preparing leaf sections (10 μm thickness) using a cryostat microtome (Leica 2800) operating at -20°C. For detection of flavonoids, sections were immersed for 15 min in Wilson’s reagent consisting of citric acid: boric acid (5/5, w/w) in 100 ml absolute ethanol, mounted in glycerol 75% (v/v) and examined using an epifluorescence microscope (NIKON, F 1200) under UV (BP 340–380 nm, LP 425 nm). Yellow fluorescence indicated the presence of flavonoids [27].

For detecting phenolics, leaf sections were placed on a glass slide, covered with a glass cover slip, and examined with the aid of an epifluorescence microscope (NIKON, F 1200) (excitation filter of 390–420 nm and an emission filter of 425–450 nm). Phenolics emitted blue fluorescence under UV light [28]. For lignin observation, leaf disks were punched from control and thiamine-treated plant leaves, boiled for 10 min in 95% ethanol and then treated with phloroglucinol (10 g in 95 ml of absolute ethanol) for 3 min. The disks were washed in 25% HCl, mounted in glycerol (75%), and examined under a light microscope [37].

### Microscopic assessment of *P*. *viticola* structures

For the observation of *P*. *viticola* mycelia growing in grapevine foliar tissues, leaf discs (1.4 cm diameter) from thiamine- or water-treated and inoculated plants were incubated at 100°C in 1 M KOH for 15 min, washed 3 times in water for 15 min, stained with 0.05% (w/v) aniline blue in 0.067 M K_2_HPO4 (pH 9–9.5) for 15 min and observed with an epifluorescence microscope (NIKON, F 1200) under UV light (excitation 340 nm, emission 380 nm, stop filter LP 430 nm) according to the method of Diez-Navajaz et al. [34]. The reaction of aniline blue with β-1,3-glucans, makes *P*. *viticola* mycelia visible under these conditions as a blue fluorescence. For each treatment, 20 leaf discs from 6 plants were randomly isolated for microscopic observations and the whole experiment was repeated 3 times.

### Statistical analysis

Data were analyzed by ANOVA using the Statistica 7.1 Software program (StatSoft, Tulsa, OK, USA). Almost, significance of the differences between mean values was determined with Tukey’s HSD test (P ≤ 0.05).

## Abbreviations

ABTS[*][+]: 2,2^′^-azinobis(3-ethylbenzothiazoline-6-sulfonic acid); COADS: Chromatographic online antioxidant detection system; DPT: Days post-treatment; DPI: Days post-inoculation; HPT: Hours post-treatment; IR: Induced resistance; Trolox: 6-hydroxy-2,5,7,8-tetramethylchroman-2-carboxylic acid.

## Competing interests

The authors declare that they have no competing interests.

## Authors’ contributions

HB conceived the study, carried out the culture, treatment and inoculation of grapevine plants, assessed the disease severity, analyzed the expression of defense related-genes by Real Time q-PCR, analyzed cellular defense responses by histochemistry, assessed *P*. *viticola* development by epifluorescence microscopy, helped to HPLC analysis of stilbenes and wrote the manuscript. AP realized HPLC-DAD analysis of stilbenoids and quercetin flavonol and helped to draft the manuscript. M-AW participated in the statistical analysis of the data and helped to draft the manuscript. CC, DS and CM realized the HPLC-AOx analysis of the total antioxidant potential of grapevine plants and identified the phenolic compounds implicated. RB realized the ULPC-MS analysis in order to confirm the identity of the stilbenoids and helped in writing the manuscript. AM and IS-G coordinated the research project and supervised the work. All authors read and approved the final manuscript.
